# The Critical Care Phenotype of Hypokalemic Paralysis: Etiology, Outcomes, and Predictors of Respiratory Failure in a Retrospective Cohort Study

**DOI:** 10.7759/cureus.103865

**Published:** 2026-02-18

**Authors:** Arunkumar R Pande, Nitin Rai, Shilpi Manchanda, Abhishek Srivastava, Sandeep Agarwal, Indu C Srivastava, Ashish Awasthi

**Affiliations:** 1 Endocrinology, Lucknow Endocrine Diabetes and Thyroid Clinic (LEDTC) and Heath City Vistaar, Lucknow, IND; 2 Critical Care Medicine, Health City Vistaar, Lucknow, IND; 3 Internal Medicine, Max Hospital, Lucknow, IND; 4 Neurology, Max Hospital, Lucknow, IND; 5 Endocrinology, Max Hospital, Lucknow, IND; 6 Toxicology and Experimental Medicine, Council of Scientific and Industrial Research-Central Drug Research Institute (CSIR-CDRI), Lucknow, IND

**Keywords:** acute flaccid quadriparesis, critical care, electrolyte disorders, hypokalemic paralysis, mechanical ventilation, respiratory failure

## Abstract

Background: Hypokalemic periodic paralysis (HPP) presenting as acute quadriparesis is a neuromuscular emergency. While its etiology is described in general wards, its severe "critical care phenotype" in the intensive care unit (ICU) remains poorly characterized. We aimed to define this phenotype by analyzing the clinical profile, etiological spectrum, and predictors of life-threatening severity.

Methods: A retrospective study was conducted of 12 patients (nine male, three female; median age: 31.5 years) admitted to a tertiary ICU (2015-2021) with acute quadriparesis and hypokalemia (median potassium: 1.75 mmol/L). We analyzed management and outcomes and compared patients requiring mechanical ventilation (MV+) with those who did not (MV−) using distribution-appropriate statistical methods to identify factors associated with respiratory failure.

Results: All patients presented with acute flaccid quadriparesis and areflexia. Five (41.7%) required invasive mechanical ventilation, defining a severe "critical care phenotype." A secondary cause was identified in eight patients (66.7%), including thyrotoxicosis (n=2), distal renal tubular acidosis (n=2), primary hyperaldosteronism, sepsis, dengue fever, and gastroenteritis. Critically, the need for mechanical ventilation was not associated with the degree of hypokalemia (MV+ 1.7 mmol/L vs. MV- 1.7 mmol/L, p=0.87) or other baseline characteristics. With potassium supplementation and targeted therapy, 11 patients (91.7%) achieved complete neurological recovery; one death occurred in a patient with sepsis.

Conclusion: HPP in the ICU represents a distinct critical care phenotype with a high risk of respiratory failure. As the requirement for mechanical ventilation was not predicted by admission potassium levels, vigilant monitoring for respiratory muscle fatigue is warranted in all cases. Favorable outcomes are achievable with prompt correction and treatment of the underlying cause, reinforcing that HPP is a reversible ICU emergency.

## Introduction

Electrolyte imbalances are a common and consequential challenge in the intensive care unit (ICU), significantly influencing morbidity and outcomes [1]. Hypokalemia is among the most frequent and clinically significant of these abnormalities [2]. Its manifestations range from nonspecific symptoms to life-threatening complications, including cardiac arrhythmias and profound neuromuscular weakness that can progress to acute flaccid quadriparesis [3]. In critically ill patients, prompt recognition and management of hypokalemia are vital to prevent these severe sequelae [4].

Hypokalemic periodic paralysis (HPP) represents a potentially life-threatening neuromuscular complication of hypokalemia. While the etiological spectrum of HPP has been described in general medical and neurological wards, a review of the literature reveals a dominance of such ward-based cohorts and isolated case reports [5,6]. Indian case series have demonstrated that a substantial proportion of adult HPP cases are secondary in nature, most commonly due to renal tubular acidosis and primary hyperaldosteronism [7]. A significant knowledge gap exists, with a notable absence of dedicated series focusing on the most severe patients requiring ICU care. Consequently, the frequency of life-threatening respiratory failure, the defining features of a 'critical care phenotype,' and the factors predicting this extreme severity remain poorly characterized.

To address this critical gap, we conducted a retrospective study of patients presenting with hypokalemic quadriparesis to our ICU. The primary objective was to characterize the clinical profile, etiological spectrum, and outcomes of this severe presentation. A key analytical objective was to identify factors associated with the highest acuity of illness, defined by the need for invasive mechanical ventilation, to better define and understand this critical care phenotype.

## Materials and methods

A retrospective observational study was conducted in the ICU of a tertiary care center in Northern India between August 2015 and August 2021. A comprehensive review of all adult ICU admissions during this period was conducted to identify potential cases by screening the clinical database and electronic medical records for any admission documented with a primary presentation of acute weakness, paralysis, or quadriparesis. The medical charts of all identified patients were then individually reviewed for eligibility based on the following criteria: inclusion required patients to be adults (≥18 years) presenting with acute flaccid quadriparesis where hypokalemia was determined to be the primary cause, a diagnosis based on established clinical criteria that include the acute onset of flaccid weakness, documented hypokalemia, and the exclusion of alternative neuromuscular disorders, with a confirmatory response to potassium repletion [8]. A single patient with a serum potassium level of 3.6 mmol/L was included, as the patient presented with the classic clinical triad, including hypokalemic electrocardiographic changes, and demonstrated rapid, confirmatory neurological recovery following potassium administration, underscoring that the diagnosis is clinical and not solely reliant on a specific potassium threshold. Patients were excluded if an alternative definitive cause for weakness (e.g., Guillain-Barré syndrome confirmed by neurophysiology, spinal cord compression on imaging) was identified or if medical records were incomplete. The following data were systematically extracted from the medical records: demographics, clinical presentation, laboratory results (including serum potassium, creatine phosphokinase, thyroid function tests, arterial blood gas, and spot urine potassium/creatinine ratio where available), need for ventilatory support, identified etiology, treatment, and outcome. The study was approved by the Sanjivini Lung Centre Ethics Committee, and the requirement for informed consent was waived due to its retrospective nature.

Diagnostic evaluation and etiological workup

All patients underwent a detailed clinical history and focused physical examination at presentation, with particular attention to features suggestive of secondary causes of hypokalemia, including symptoms of thyrotoxicosis, hypertension, gastrointestinal losses, systemic illness, and prior similar episodes. A uniform laboratory evaluation was performed on all patients, including serum electrolytes, arterial blood gas analysis for acid-base status, thyroid function tests, and additional biochemical tests as clinically indicated. Etiological evaluation was conducted using a stepwise diagnostic approach for hypokalemia, consistent with standard algorithms described in Harrison’s Principles of Internal Medicine, to differentiate transcellular potassium shifts from renal and extrarenal potassium losses [9,10]. Targeted confirmatory investigations, including imaging studies, were performed selectively when guided by clinical and biochemical findings, such as adrenal imaging in patients with laboratory evidence suggestive of primary hyperaldosteronism.

Statistical analysis was performed using Stata version 19.5 (StataCorp. 2025. Stata Statistical Software: Release 19. College Station, TX: StataCorp LLC). Continuous, non-normally distributed variables are presented as median (interquartile range) and were compared using the Mann-Whitney U test. Categorical variables were presented as counts (percentages) and compared using Fisher's exact test; p-values < 0.05 were considered statistically significant.

## Results

Cohort characteristics

We identified 12 patients who met the inclusion criteria for HPP requiring ICU care. The cohort had a median age of 31.5 years (IQR 23.5-39.5) with a strong male predominance (9:3 ratio). Clinically, all patients presented with acute flaccid quadriparesis and areflexia. The severity of illness was notable, with five patients (41.7%) requiring invasive mechanical ventilation. Prodromal symptoms were reported in a minority, including myalgia (n=3), fever (n=3), and gastrointestinal symptoms (n=2) (Table 1).

**Table 1:** Cohort Characteristics and Clinical Outcomes of Patients With Hypokalemic Paralysis (N=12). Data are presented as median (interquartile range) for continuous variables and as number (percentage) for categorical variables.

Variable	Summary Statistic
Demographics	
Age, years	31 (23.5–39.5)
Sex, Male: Female	9:3
Disease Severity	
Serum Potassium on Admission, mmol/L	1.75 (1.55–2.15)
Required Mechanical Ventilation, n (%)	5 (41.7%)
Etiology	
Cases with an Identified Secondary Cause, n (%)	8 (66.7%)
Specific Secondary Causes, n	
Thyrotoxic Periodic Paralysis	2
Distal Renal Tubular Acidosis (dRTA)	2
Primary Hyperaldosteronism	1
Sepsis	1
Dengue Fever	1
Gastroenteritis	1
Outcomes	
Complete Neurological Recovery, n (%)	11 (91.7%)
Mortality, n (%)	1 (8.3%)

Profound hypokalemia was a universal feature at presentation, with a median serum potassium of 1.75 mmol/L (IQR 1.55-2.15). One patient was included with a borderline potassium level of 3.6 mmol/L based on the presence of the classic clinical triad and a confirmatory response to potassium repletion, consistent with established diagnostic criteria [7]. Detailed clinical profiles of all patients are provided in Table 2.

**Table 2:** Clinical and Laboratory Profile of the 12 Patients With Hypokalemic Periodic Paralysis. Data are presented as absent (-) or present (+), unless otherwise specified. CPK: creatine phosphokinase; dRTA: distal renal tubular acidosis; NR: not recorded; HPP: hypokalemic periodic paralysis.

Parameter	Case 1	Case 2	Case 3	Case 4	Case 5	Case 6	Case 7	Case 8	Case 9	Case 10	Case 11	Case 12
Demographics & History												
Age (years)	23	24	26	34	28	41	70	35	22	56	20	38
Sex	M	M	M	F	M	F	M	M	M	M	M	F
Previous attacks	-	-	-	-	-	-	-	-	-	-	-	-
Family history	-	-	-	-	-	-	-	-	-	-	-	-
Presenting Illness												
Symptom duration (days)	5	3	NR	2	5	NR	NR	1	NR	NR	NR	1
Fever	-	-	+	-	+	+	-	-	-	+	-	-
Gastrointestinal loss	-	-	-	-	+	-	-	-	-	-	-	-
Myalgia	+	-	+	-	-	-	-	-	-	-	-	-
Severity & Examination												
Required ventilatory support	+	+	-	-	+	+	-	+	-	-	-	-
Areflexia	+	+	+	+	+	+	+	+	+	+	+	+
Investigations												
Serum K+ (mmol/L)	1.3	2.2	1.8	1.5	1.8	1.6	1.6	1.7	2.1	1.5	3.6	1.7
CPK elevated	-	-	-	-	+	-	+	-	+	-	-	+
Outcome & Etiology												
Outcome	Full recovery	Expired	Full recovery	Full recovery	Full recovery	Full recovery	Full recovery	Full recovery	Full recovery	Full recovery	Full recovery	Full recovery
Final Etiological Diagnosis	Graves' disease	Idiopathic HPP	Dengue fever	Idiopathic HPP	Gastroenteritis	Idiopathic HPP	dRTA	dRTA	Thyroiditis	Sepsis	Idiopathic HPP	Primary hyperaldosteronism

Etiology, management, and outcomes

A systematic diagnostic workup revealed a secondary cause for HPP in eight patients (66.7%). The etiological spectrum was diverse, including thyrotoxic periodic paralysis (n=2), distal renal tubular acidosis (dRTA, n=2), and single cases of primary hyperaldosteronism (biochemically confirmed with suppressed renin and elevated aldosterone), sepsis, dengue fever, and gastroenteritis (Table 1).

Management centered on aggressive intravenous potassium supplementation, combined with targeted therapy for the underlying etiology (e.g., antithyroid drugs, potassium-sparing agents, mineralocorticoid antagonists). The outcome was favorable, with 11 patients (91.7%) achieving complete neurological recovery. The single mortality occurred in a patient with sepsis and concomitant acute kidney injury.

Analysis of the critical care phenotype

To further characterize the severe 'critical care phenotype,' we compared patients who required mechanical ventilation (MV+, n=5) to those who did not (MV-, n=7). As detailed in Table 3, the Mann-Whitney U test found no significant difference in the median serum potassium level at presentation (MV+ 1.7 mmol/L, IQR 1.6-1.8 vs. MV- 1.7 mmol/L, IQR 1.5-2.1; p=0.87). Similarly, Fisher’s exact test showed no significant differences in the distribution of sex, the prevalence of a secondary etiology, fever, or an elevated creatine phosphokinase level between the groups (all p > 0.05). The only recorded mortality occurred in the MV+ group. This analysis indicates that within our cohort, the development of respiratory failure was not predicted by these baseline demographic or admission laboratory parameters.

**Table 3:** Comparison of Patients Requiring vs. Not Requiring Mechanical Ventilation.

Variable	Mechanical Ventilation (n=5)	No Mechanical Ventilation (n=7)	Test Statistic	P-value
Age, years	34 (26–41)	34 (22–56)	U = 16.0	0.94
Sex, Male	5 (100%)	5 (71.4%)	Fisher's Exact	0.51
Serum K+, mmol/L	1.7 (1.6–1.8)	1.7 (1.5–2.1)	U = 15.5	0.87
Etiology, Secondary	4 (80%)	4 (57.1%)	Fisher's Exact	0.59
Presence of Fever	2 (40%)	1 (14.3%)	Fisher's Exact	0.53
CPK Elevated	2 (40%)	1 (14.3%)	Fisher's Exact	0.53
Mortality	1 (20%)	0 (0%)	Fisher's Exact	0.38

The current analysis represents a comprehensive, single-center case series focusing on the critical care outcome of hypokalemic paralysis. The clinical details of a patient from this cohort (Case 9) have been previously reported in a smaller series focused on the etiological spectrum [11]. Furthermore, a preliminary analysis of 10 cases, which included an overlapping subset of the present cohort, was presented as an abstract [12]. The present manuscript provides a novel characterization of the 'critical care phenotype,' includes new cases, and presents a complete analysis of management and outcomes that is not available in prior publications.

## Discussion

Hypokalemic quadriparesis is a rare but potentially life-threatening clinical syndrome, while its etiological spectrum is well-documented in general inpatient settings [5,6]. Its manifestation as a 'critical care phenotype' requiring ICU admission remains poorly characterized. We describe 12 consecutive cases of HPP presenting with acute flaccid quadriparesis to the ICU, defining a severe presentation marked by a high rate of life-threatening respiratory failure (5/12 patients requiring mechanical ventilation). This acuity likely reflects a confluence of profound hypokalemia and systemic triggers like sepsis and thyrotoxicosis, underscoring that in the critically ill, hypokalemic paralysis is often part of a broader physiological crisis rather than an isolated electrolyte disturbance.

Despite this extreme severity, the underlying etiological spectrum remains diverse, and most importantly, our outcomes prove that with aggressive supportive care and a simultaneous, systematic search for the underlying etiology, complete neurological recovery is the expected outcome. These findings support the characterization of hypokalemic paralysis as a reversible emergency in the ICU and highlight the importance of maintaining a high index of suspicion for impending respiratory failure.

A pivotal finding of our analysis was that the need for mechanical ventilation was not predicted by the absolute serum potassium level, demographic factors, or the presence of a secondary etiology (Table 3). This suggests that the 'critical care phenotype' is driven more by the acuity and velocity of the intracellular potassium shift, or by individual neuromuscular susceptibility, than by these baseline parameters. This observation challenges the conventional reliance on a single potassium value for risk stratification and mandates vigilant respiratory monitoring in all patients presenting with severe weakness, irrespective of their initial laboratory values. Although no individual clinical or biochemical variable predicted the need for mechanical ventilation, patients requiring ventilatory support uniformly presented with severe neuromuscular manifestations, including profound hypokalemia and generalized areflexia. From a mechanistic perspective, secondary causes such as renal tubular acidosis, characterized by sustained potassium loss and metabolic acidosis, may plausibly contribute to severe weakness during acute episodes.

When comparing our etiological spectrum with the existing literature, our finding of a high rate of secondary causes (66.7%) aligns with global trends. Garg et al. identified a secondary etiology in 57.7% of 29 patients in a neurology setting [13]. Additionally, a larger study from Taiwan reported secondary causes in 68% of patients, with thyrotoxicosis being the most frequent [14]. The spectrum of secondary causes in our ICU cohort, including thyrotoxicosis, renal tubular acidosis, and primary hyperaldosteronism, mirrors this diversity, indicating that the path to severe paralysis is multifactorial. Other rare causes of secondary HPP, such as Liddle's syndrome, were considered in the differential diagnosis but were not identified in the present cohort.

Although hypokalemic paralysis has been well described in general medical and neurological wards, studies specifically examining patients requiring ICU admission remain scarce, with most published data limited to ward-based cohorts and isolated case reports.

Our experience confirms the importance of identifying precipitating factors, a principle emphasized in large thyrotoxic periodic paralysis studies [8,15]. While we identified specific triggers such as fever and heavy exertion in a quarter of our cohort (3/12 patients), this likely reflects the challenge of ascertaining triggers in acute, life-threatening presentations. Nevertheless, when a trigger is identified, advising on its avoidance remains a cornerstone of preventing recurrence [16].

The diverse etiologies in our cohort, ranging from transcellular shifts in thyrotoxicosis to renal losses in tubular disorders, underscore the multiple pathophysiological pathways converging on severe hypokalemic paralysis [17]. This is exemplified by ongoing debates about specific triggers, such as the dengue virus, which may act as an acute precipitant in susceptible individuals rather than a direct cause [18].

The diagnosis of HPP rests on the clinical triad of acute flaccid weakness, hypokalemia, and the exclusion of alternatives. Its most critical mimic is Guillain-Barré syndrome, from which it can be distinguished by its dramatically rapid progression and resolution with potassium repletion [16,19,20]. This temporal profile is a crucial diagnostic clue. A systematic workup, guided by acid-base status and other clinical clues, is paramount to identify the underlying etiology, as demonstrated by our successful identification of diverse causes [21].

The cornerstone of acute management is potassium supplementation, with intravenous administration reserved for severe manifestations such as respiratory paralysis or arrhythmias, as employed in the majority of our patients (22). Crucially, concurrent management of the underlying etiology is essential to prevent recurrence and ensure sustained recovery, including antithyroid drugs for thyrotoxic periodic paralysis, potassium and alkali supplementation for renal tubular acidosis, and mineralocorticoid receptor antagonists for primary hyperaldosteronism [15,22,23]. Patients require close monitoring for rebound hyperkalemia following correction.

Our study has several strengths, including its focus on the underreported critical care population of hypokalemic paralysis and the demonstration that a broad etiological spectrum is present even among the most severely affected patients. However, these findings must be interpreted considering certain limitations. The retrospective, single-center design and the small sample size inherent to an ICU-based cohort limit the generalizability of our results. Due to the retrospective nature of the study, the direction of progression of weakness (ascending versus non-ascending) was not consistently documented in all patients, precluding reliable estimation of the proportion presenting with an ascending pattern of paralysis. The high acuity observed is undoubtedly influenced by referral bias, as our tertiary care center manages the most severe regional cases. Furthermore, the lack of long-term follow-up data prevents us from commenting on recurrence rates. Future prospective multicenter studies involving multiple ICUs would be valuable for further characterizing this critical care phenotype and validating our findings.

## Conclusions

Acute hypokalemic paralysis represents a treatable cause of quadriparesis that, in the ICU, may present as a severe critical care phenotype with a significant risk of respiratory failure. A systematic evaluation for underlying causes is essential, as most cases have a secondary and potentially reversible etiology. With prompt potassium repletion and targeted therapy, favorable neurological recovery is achievableOur data confirm that the decision to initiate mechanical ventilation must be guided by vigilant clinical monitoring for signs of respiratory muscle fatigue, rather than any specific potassium threshold, thereby solidifying this condition as a quintessential reversible emergency in the ICU.
